# Enhanced fatty acid oxidation in osteoprogenitor cells provides protection from high-fat diet induced bone dysfunction

**DOI:** 10.1093/jbmr/zjae195

**Published:** 2024-12-08

**Authors:** Ananya Nandy, Ron C M Helderman, Santosh Thapa, Sun H Peck, Alison Richards, Shobana Jayapalan, Nikita Narayani, Michael P Czech, Clifford J Rosen, Elizabeth Rendina-Ruedy

**Affiliations:** Division of Clinical Pharmacology, Department of Medicine, Vanderbilt University Medical Center, Nashville, TN 37232, United States; Division of Clinical Pharmacology, Department of Medicine, Vanderbilt University Medical Center, Nashville, TN 37232, United States; Frank H. Netter M.D. School of Medicine, Quinnipiac University, North Haven, CT 06518, United States; Division of Clinical Pharmacology, Department of Medicine, Vanderbilt University Medical Center, Nashville, TN 37232, United States; Division of Clinical Pharmacology, Department of Medicine, Vanderbilt University Medical Center, Nashville, TN 37232, United States; Department of Biochemistry, Vanderbilt University School of Medicine, Nashville, TN 37232, United States; Department of Biomedical Engineering, Vanderbilt University School of Engineering, Nashville, TN 37232, United States; Department of Veterans Affairs, Nashville Veterans Affairs Medical Center, Tennessee Valley Healthcare System, Nashville, TN 37232, United States; Division of Clinical Pharmacology, Department of Medicine, Vanderbilt University Medical Center, Nashville, TN 37232, United States; Division of Clinical Pharmacology, Department of Medicine, Vanderbilt University Medical Center, Nashville, TN 37232, United States; Division of Clinical Pharmacology, Department of Medicine, Vanderbilt University Medical Center, Nashville, TN 37232, United States; Program in Molecular Medicine, University of Massachusetts Chan Medical School, Worcester, MA 01655, United States; Maine Health Institute for Research, Scarborough, ME 04074, United States; Division of Clinical Pharmacology, Department of Medicine, Vanderbilt University Medical Center, Nashville, TN 37232, United States; Molecular Physiology and Biophysics, Vanderbilt University, Nashville, TN 37232, United States

**Keywords:** lipids, metabolism, diabetes, fracture, perilipin 2

## Abstract

Bone homeostasis within the skeletal system is predominantly maintained by bone formation and resorption, where formation of new bone involves maturation of stromal cells to mineral and matrix secreting mature osteoblasts, which requires cellular energy or adenosine triphosphate. Alterations in systemic metabolism can influence osteoblast function. In line with this, type 2 diabetes mellitus (T2DM), a common metabolic disorder is also associated with reduced bone formation and increased risk of fracture. Impairment in lipid metabolism is one of the key features associated with T2DM-related pathologies in multiple tissues. Therefore, we tested the hypothesis that the reduced bone formation reported in obese murine models of impaired glucose tolerance is a function of disrupted lipid metabolism in osteoblasts. We first confirmed that mice fed a high-fat diet (HFD) have reduced bone microarchitecture along with lower bone formation rates. Interestingly, osteoblasts from obese mice harbor higher numbers of cytosolic lipid droplets along with decreased bioenergetic profiles compared to control cells. Further supporting this observation, bone cortex demonstrated higher total lipid content in HFD fed mice compared to control-fed mice. As a further proof of principle, we generated a novel murine model to conditionally delete *Plin2* in osteoblast-progenitor cells using *Prrx1*-Cre, to enhance lipid droplet breakdown. Our data demonstrate that knocking down *Plin2* in an osteoprogenitor specific manner protects from HFD induced osteoblast dysfunction. Furthermore, the mechanism of action involves enhanced osteoblast fatty acid oxidation. In conclusion, the current studies establish that HFD induced glucose intolerance leads to perturbations in osteoblast lipid metabolism, thus causing lower bone formation, which can be protected against by increasing fatty acid oxidation.

## Graphical Abstract

**Graphical Abstract:**
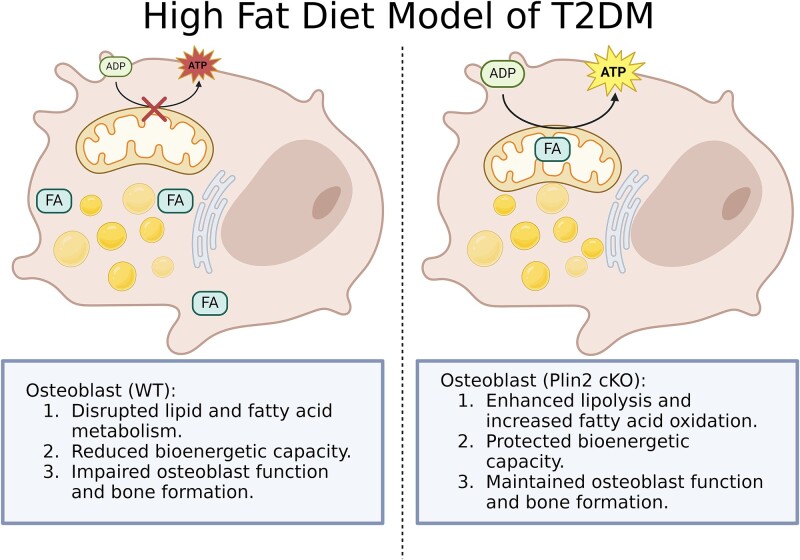

## Introduction

Bone is a highly dynamic tissue with simultaneous interplay between two energy consuming processes, formation of new bone by osteoblasts and bone resorption or removal of old bone by osteoclasts.^1^^,^^2^ Formation of new bone is associated with endergonic reactions that require energy to support secretion of extracellular matrix and mineralization.^3^^,^^4^ These processes require constant supply of biofuel generated from the breakage of high energy phosphate bonds, as in adenosine triphosphate (ATP). Metabolic disorders are expected to influence the supply of cellular ATP, which can have a direct impact on cellular function. Within the context of the osteoblast, these alterations in energy production are likely to negatively impact bone formation and skeletal homeostasis. However, this is an understudied area within the musculoskeletal field.

Type 2 diabetes mellitus (T2DM) is a complex metabolic disorder that results in hyperglycemia and insulin resistance that affects almost every organ in the human body including the skeletal system.^5–9^ Interestingly, T2DM is also closely associated with impaired lipid metabolism where 80%-90% of T2DM patients are either overweight or obese.^10^^,^^11^ Impaired lipid metabolism in T2DM causes accumulation of ectopic lipid accumulation in several tissues that further leads to dysfunction of multiple organs, which include hypertrophy of adipose tissue,^12^^,^^13^ hepatic steatosis,^14^^,^^15^ as well as intra- and intermyocellular lipid accumulation.^16^ Recently, our lab demonstrated that cytosolic lipolysis regulated by adipose triglyceride lipase (ATGL) is required for proper osteoblast function.^17^ In this capacity, small lipid droplets are present in osteoblasts^18^ and serve as a source of fatty acid substrates during mildly nutrient deplete conditions such as overnight fasting. Importantly, these lipid droplets directly influence osteoblast bioenergetic status, such that when fatty acids are unavailable via impaired-lipolysis, osteoblast activity and subsequent bone formation is reduced.^17^ Given these data, along with others that have demonstrated the importance of fatty acid metabolism for bone formation,^19–21^ we sought to further characterize osteoblast lipid metabolism in a diet-induced obesity model of T2DM. We further aimed to generate an osteoprogenitor specific knock out of perilipin 2 (PLIN2) to enhance lipolysis as a mechanism to protect from T2DM associated bone dysfunction. We have previously reported that *Plin2* is the most abundant isoform of perilipin expressed in osteoblasts,^18^ and others have used similar murine-model systems to protect from diet-induced obesity tissue dysfunction.^22^^,^^23^ Therefore, the current project investigates whether osteoblast lipid dysfunction contributes to skeletal fragility observed in T2DM.

## Materials and methods

### Animal models

High-fat diet (HFD) studies included 5-wk old C57BL6/N male mice that were fed a control (10 kcal% fat, sucrose-matched; Research Diets; D12450J) or HFD (60 kcal% fat; Research Diets; D12492) for 8 wk as we have previously characterized this to be an appropriate time point to induce impaired glucose tolerance.^24–27^ For conditional knock-out studies, Plin2^tm1a(EUCOMM)Wtsi^ (Plin2^fl/fl^) embryos were purchased from Infrafrontier (EMMA ID: EM: 05522) and implanted into 2 pseudopregnant, C57BL/6N females. Offspring were then mated with B628S4-Gt(ROSA)26Sor^tm1(FLP1)Dym^ (Cat#11065, Jackson Laboratory, Bar Harbor, ME) to generate the floxed *Plin2* allele with removal of the neomycin resistant (Neo) cassette. This breeding schematic has previously been described and utilized by Najt et al.,^28^ to generate Plin2^fl/fl^ mice. The resulting Plin2^fl/fl^ mice were then bred with our Prx1-Cre (Prx1-Cre^TG/+^) which have been deposited at the Jackson Laboratories (Strain#: 033173), to generate Prx1-Cre.Plin2^fl/fl^ (ΔPlin2) mice along with Cre negative littermate controls, Plin2^fl/fl^ (Control). Again, at 5 wk of age these mice were fed either a high fat (60 kcal% fat, Research Diets; D12492) or matched sucrose control diet (10 kcal% fat, Research Diets; D12450J) and maintained on this diet for 12 wk. Mice for both studies were maintained on a standard 12-hr light/dark cycle and had access to ad libitum food and RO water. Standard housing conditions are within a range of 30%-70% humidity with a set point of 50% and a temperature range of 68-76 °F with a set point of 72 °F. Mice were housed 3-4 mice per cage, from the same litters, and had access to enrichment huts. The animal’s environment and all procedures performed were in accordance with the Institutional Animal Care and Use Committee at Vanderbilt University Medical Center.

### Bone marrow stromal cell isolation and osteoblast cell culture

As previously described,^29^ primary murine bone marrow stromal cells (BMSCs) were collected from the long bones. Briefly, soft adhering tissue was carefully dissected from the femur and tibia, with the distal and proximal ends cut to expose the marrow shaft. Bone marrow was isolated by centrifugation (1 min at 10 000 rpm) and plated with complete α-MEM (Sigma; M0450), 10% fetal bovine serum (FBS) (Avantor; 89510-186), 1% penicillin/streptomycin (Sigma; P4333) and maintained in 5% CO_2_ at 37 °C. After 48 hr the non-adherent hematopoietic cells were discarded, while the adherent BMSC population was trypsinized, counted and plated according to targeted outcomes, detailed within each assay. Osteogenic differentiation was accomplished using complete α-MEM, supplemented with 5 mM β-glycerol phosphate (Sigma; G9422) and 50 μg/mL ascorbic acid (Sigma; A4544).^29^

### Immunostaining, imaging, and analysis

BMSCs were seeded on collagen-coated coverslips (1.75 × 10^5^ cells/mL) and cultured with osteogenic media. At the end of experiment, cells were fixed with neutral buffered, methanol-free, 4% formaldehyde for 20 min. Cells were washed in 1× phosphate buffered saline (PBS) three times and neutral lipids were stained using 10 μM BODIPY 493/503 (Thermo Fisher; D-3922) solution and mounted with Prolong Glass Antifade Mountant with NucBlue (Thermo Fisher; P36981). Confocal Z stack images were taken with a Zeiss LSM 880 at 0.30 mm sections. Image J software was used to identify and count lipid droplets, as well as measure the intensity and size.

### RNA isolation

Total RNA was extracted from the femur cortex (no marrow) that were flash frozen and pulverized under liquid nitrogen using a Freezer Mill (Spex Sample Prep). RNA isolation was accomplished using Trizol according to the manufacturers protocol and subsequently further purified (RNeasy MinElute Cleanup Kit; 74204). RNA from adipose tissue was isolated using Trizol and a handheld homogenizer (Fisher; Homogenizer 850). The RNA quality and quantity were assessed by spectrophotometry (Nanodrop One, Thermo Fisher).

### Gene expression analysis

Complimentary DNA (cDNA; 12.5 ng) was reverse transcribed from purified RNA (Thermo Fisher; 4374967). Expression of selected target genes was analyzed using quantitative real-time polymerase chain reaction (qRT-PCR) with SYBR green chemistry (Thermo Fisher; PowerUP SYBR Green; A25742) on a QuantStudio 5 (Thermo Fisher). All target genes were normalized to the housekeeping gene *Hprt* and 2^−ΔΔCq^ method was used to express data.^30^ Gene sequences can be found in Table S1.

### Protein abundance

Homogenized flash frozen femur cortex, devoid of marrow elements was used to harvest total protein. Total protein was extracted by resuspending the powdered bone in 1× RIPA buffer (Cell Signaling Technology; 9806) containing PMSF (1 mM, Cell Signaling Technology; 8553S) and sufficient mechanical disruption. Protein concentration was determined by BCA assay, and 30 μg of protein was probed for PLIN2 using sodium dodecyl sulfate-polyacrylamide gel electrophoresis (SDS-PAGE), followed by immunoblotting using anti-PLIN2 antibodies (Novus Biologicals; 40877). Target protein was normalized against total protein abundance using No Stain Protein Labeling Reagent (Thermo Fisher Scientific; A44717).

### Seahorse flux assays

To measure kinetic metabolic flux, a Seahorse Flux analyzer was used along with respective assays. BMSCs were seeded in Seahorse XFe 96-well plates at 2.5 × 10^4^ cells/well and were cultured under osteogenic conditions as described above.^31^^,^^32^

#### Mitochondrial stress test and ATP production

To measure the mitochondrial ATP production of differentiated osteoblasts, “Mito Stress Test” was performed according to the manufacturers protocol (Agilent; 103015-100) with minor deviations. Briefly, on the day of the assay cell; culture media was removed and cells washed with basal assay Dulbecco's Modified Eagle Medium (DMEM) medium (Agilent; 103575-100) containing 10 mM glucose (Agilent; 103577-100), 2 mM glutamine (Agilent; 103579-100), 1 mM sodium pyruvate (103578-100), 200 nM insulin and 60 μM oleic acid-BSA (Sigma; O3008). During the assay, sequential injections of 2 μM oligomycin A (Sigma; 75351), 2 μM FCCP (Sigma; C2920), and 1 μM rotenone/ 1 μM antimycin A (Sigma; R8875, A8674) were used to analyze the effect on mitochondrial function and calculate mitochondrial ATP production. Real time oxygen consumption rates (OCR) and extracellular acidification rates (ECAR) were measured throughout the assay.

#### Cell energy phenotype

To measure metabolic potential, “Cell Energy Phenotype Test” was performed according to the manufacturers protocol (Agilent; 103325-100). Briefly, baseline OCR and ECAR were measured followed by a simultaneous injection of 2 μM oligomycin A and 2 μM FCCP (Sigma; C2920) to determine metabolic potential under “normal” conditions and when “stressed”.

For all seahorse assays, data have been normalized per cell number. Cell counting was accomplished by injecting Hoechst 33342 stain (Thermo Scientific; 62249) into the wells during the last injection, and automated cell counting was performed using a Cytation 5 (BioTek).

### Dual-energy X-ray absorptiometry

Body composition was monitored over time by dual-energy X-ray absorptiometry (DXA) (Faxitron UltraFocus, Hologic) in anesthetized mice placed in the prone position. The DXA was calibrated for dark images, offset images, and flat-field images before the measurement by the manufacturer's provided method.

### Micro-computed tomography

Femurs were stored in PBS at −20 °C while vertebra were fixed in 10% neutral-buffered formalin (NBF) for 48 hr, then stored in 70% ethanol. Bone microarchitecture was determined using micro-computed tomography (μCT 50, Scanco Medical AG, Brüttisellen, Switzerland). Scans of the femur were obtained from the distal femur metaphysis and mid-diaphysis portions for analysis of the trabecular and cortical bone, respectively. Vertebral scans were performed on the L6 vertebral body. All scans were performed with an X-ray tube peak intensity at 70 kVp, 114 mA, and an integration time of 300 ms. Femur distal metaphysis and mid-diaphysis image stacks were taken with an isotropic voxel size of 6 μm. Femur analyses were performed for trabecular bone by selecting a standardized region of interest (ROI) 270 μm above the peak of the distal growth plate as to include a region of trabecular bone that extends proximally over 2.6 mm. Trabecular bone of the vertebra was scanned with an isotropic voxel size of 9 μm, and analysis included ROI defined as secondary spongiosa within L6. Trabecular bone analysis of the femur and vertebra was performed with Gaussian image filtering (support of 1.0 and sigma of 0.1) and at a threshold of 350 mgHA/cm^3^. Cortical bone was analyzed in a 0.5 mm-long region (50 transverse slices) at the femoral mid-diaphysis with the same image filtering but at a threshold of 435 mgHA/cm^3^.

### Lipid isolation from bone, thin layer chromatography and mass spectrometry analysis

Total lipids were extracted from tibia cortex (bone marrow removed via centrifugation) using the Bligh and Dyer method.^33^ Following pulverization, samples were weighed before adding chloroform:methanol (1:2) mix for normalization and kept overnight in chloroform:methanol in a 37 °C water bath for more efficient extraction. The desiccated lipid extract was resuspended in an equivalent volume of chloroform:methanol (2:1) mixture (80 μL) and 10 μL was loaded onto a thin layer chromatography (TLC) plate (Sigma; 1.05553.0001). Lipids underwent separation through TLC development at 4 °C in a solvent of hexanes:diethyl ether:acetic acid (70:30:1) (Sigma; 293253, Emparta; 1.07026.2500, Sigma; 695092). Staining of TLCs was done in 10% (wt/vol) copper sulfate in an 8% (vol/vol) phosphoric acid solution, followed by charring at 120 °C to visualize the lipids. Analysis of the lipid bands was conducted using Fiji software, where the integrated density of lipid bands was normalized to initial bone powder weight.

For free long chain fatty acid (LCFA) analysis, dry mouse bone powder following liquid nitrogen pulverization was resuspended in 125 μL of 0.3 M pyridine buffer, pH 4.5. The suspension was spiked with 10 μL of internal standards mix (25 μg/mL each C16:0-d4, C22:0-d4 and C24:0-d4) and carboxylic groups were derivatized with 25 μL of Girard-T reagent (50 mg/mL in pyridine buffer) in the presence of coupling reagent (150 mg/mL EDC). Reaction mix was incubated for 1 hr at room temperature, and the excess of derivatizing agent was quenched with 0.5 M glucose. LCFA-hydrazides were extracted with 300 μL methyl *tert*-butyl ether, dried under stream of nitrogen, reconstituted in 200 μL methanol/ethanol mix (80:20, v/v), and 10 μL were analyzed by LCMS. Analysis was performed using Quantum mass spectrometer (Thermo) interfaced to a Waters Acquity UPLC system (Waters Corp., Milford, MA). An Acquity BEH-C18 reverse phase column (2.1 × 50 mm, 1.7 μm, Waters, Milford, MA) was used for all chromatographic separations. Mobile phases were made up of 0.2 % formic acid in (A) methanol/water (55:45) and in (B) acetonitrile/isopropanol (3:1). Gradient conditions were as follows: 0-2 min, *B* = 0%; 2-8 min, *B* = 0-100%; 8-10 min, *B* = 100%; 10-10.5 min, *B* = 100%-0%; 10.5-15 min, *B* = 0%. The flow rate was maintained at 300 μL/min; the total chromatographic run time was 15 min.

The triple quadrupole mass spectrometer was operated with an electrospray ionization (ESI) source in positive mode. Quantitation was based on multiple reaction monitoring (MRM) detection (see Table S2 for MRM transitions, collision energy and dwell time). The following optimized source parameters were used for the detection of analyte and internal standards: sheath gas 40 psi; auxiliary gas 5 psi; spray voltage 4.0 kV; capillary temperature 300 °C; capillary offset was set to 35. Calibration curves were constructed for by plotting peak area ratios (analyte/internal standard) against analyte concentrations for a series of ten calibrants, ranging in concentration from 0.02 to 100 μg/mL. A weighting factor of 1/Ct^2^ was applied in the linear least-squares regression analysis to maintain homogeneity of variance across the concentration range (% error ≤ 20% for at least four out of every five standards).

Acylcarnitine analyses were carried out using a Vanquish ultrahigh performance liquid chromatography (UHPLC) system interfaced to a Q Exactive HF quadrupole/orbitrap mass spectrometer (Thermo Fisher Scientific) operating in data-dependent mode. Sample extracts (10 μl) were injected in positive ESI mode. Chromatographic separation was performed using reverse-phase Kinetex C18 analytical column (2.1 mm × 100 mm, 1.7 mm particle size, Phenomenex, Torrance, CA). The column and autosampler tray temperatures were maintained at 50 and 5 °C respectively. Mobile phases were made up of 0.2% HCOOH in (A) H2O/CH3CN/CH3OH (3:1:1) and (B) CH3CN/iPrOH (1:1). Gradient conditions were as follows: 0-1 min, *B* = 0%; 1-8 min, *B* = 0-95%; 8-10 min, *B* = 95%; 10-10.5 min, *B* = 95%-0%; 10.5-15 min, *B* = 0%. The flow rate was maintained at 300 μL/min; a software-controlled divert valve was used to transfer eluent from 0 to 2.0 min and from 10.5 to 15 min of each chromatographic run to waste. The total chromatographic run time was 15 min. Mass spectra were acquired over a precursor ion scan range of *m*/*z* 200 to 1600 at a resolving power of 60 000 using the following HESI-II source parameters: spray voltage 3.5 kV; capillary temperature 250 °C; S-lens RF level 60 V; nitrogen sheath gas 40; nitrogen auxiliary gas 10; auxiliary gas temperature 300 °C. Mass spectrometry (MS)/MS spectra were acquired for the top-seven most abundant precursor ions with an MS/MS automatic gain control (AGC) target of 1e5, a maximum MS/MS injection time of 100 ms, and a normalized collision energy of 30. Specificity of detection was confirmed by monitoring fragment of *m*/*z* 85.03. Calibration curves were constructed for acylcarnitines C16:0, C18:0, C18:1, and C18:2 by plotting peak area ratios (analyte/internal standard) against analyte concentrations for a series of ten calibrants, ranging in concentration from 1 to 100 ng/mL (Table S3). A weighting factor of 1/Ct^2^ was applied in the linear least-squares regression analysis to maintain homogeneity of variance across the concentration range (% error ≤ 20% for at least 4 out of every 5 standards).

### Histology

White adipose tissue was isolated and fixed in 10% NBF for 48 hr, before storage in 70% ethanol. Samples were sequentially dehydrated using serial concentrations of ethanol solutions and paraffin embedded. Sections (5 μm) were stained with H&E. BioQuant Osteo 2018 software (version 18.2.6; BioQuant Image Analysis Corporation, Nashville, TN) was used to assess number and volume of adipocytes.

### Bone histomorphometry

Mice were injected with calcein (10 mg/kg body weight) and alizarin (1,2-dihydroxyanthraquinone) (30 mg/kg body weight) 7 and 2 d prior to sacrifice, respectively, to assess dynamic bone changes over a 5-d interval. At sacrifice, tibias were cleaned and fixed in NBF for 48 hr. They were then dehydrated using sequential acetone concentrations, and infiltrated by destabilized methlymethacrylate (90%), benzoyl peroxide (0.05%) and dibutylphthalate (10%) for 3 d at 4 °C. The solutions were changed to allow the above chemicals to be at concentrations of 85%, 15%, and 4%, respectively, for 3-4 d. Following infiltration, tibias were embedded in methylmethacrylate (MMA) and allowed to polymerize. Samples were sectioned (5 μm) in the traverse tibia plane. ROI was defined as 100 μm distal from the proximal growth plate to include metaphyseal region of 800 μm, excluding the cortical bone. Dynamic parameters such as MS/BS, MAR, and BFR/BS were measured by analyzing the fluorescent double labels. Serial sections were stained with tetrachrome and von Kossa for analysis of osteoblasts and osteoclasts within the ROI. Osteoblasts were defined as plump, cuboidal cells that lined trabecular bone (>3 cells). Osteoclasts were defined as large, multinucleated cells on the trabecular bone surface. Bone marrow adipocytes were visulaized as empty “ghost cells”, excluding vasculature or sectioning artifacts. All analyses were completed with BioQuant Osteo 2018 version 18.2.6 (BioQuant Image Analysis Corporation, Nashville, TN). Parameters and region of analyses were within the guidelines of the nomenclature committee of the American Society of Bone and Mineral Research (ASBMR).^34^^,^^35^

### Serum lipid analyses

At sacrifice, blood was collected from the carotid artery and allowed to clot at room temperature for 30 min prior to centrifugation at 4 °C at 3000 rpm for 10 min to isolate serum. Isolated serum was submitted to the Vanderbilt Analytical Service Core for further lipid analysis. Serum triglyceride and cholesterol levels were measured using standard enzymatic assays. Free fatty acids (FFAs) were measured using an enzymatic kit from Fujifilm Healthcare Solutions (HR Series NEFA-HR).

### Glucose tolerance tests

Following a 6 hr fast, mouse tails were nicked with a scalpel blade, and AlphaTRAK2 glucometer was used to measure baseline blood glucose. Following this baseline measurement, a sterile glucose solution (2 mg/kg BW) was injected in the mice (intraperitoneally) and blood glucose was measured at 15, 30, 60, 90, and 120 min. Area under the curve (AUC) was calculated for each mouse.

### Transmission electron microscopy

Tibia were de-calcified and fixed in 2% glutaraldehyde, 0.15 M EDTA, 1 M sodium cacodylate, and then washed 3 times for 20 min each in wash buffer, followed by post-fixation in 1% osmium tetroxide (60 min). After fixation, the tibias were washed 3 times for 20 min each with buffer and then dehydrated by passing through multiple ethanol dehydration steps (e.g., 50%, 70%, 80%, 90%, 95%, and 100% for 3 changes). Tibia were then washed three times for 20 min each in propylene oxide. Tissues were infiltrated by 1:1 propylene oxide and Poly/Bed resin for 5 d. Propylene oxide was allowed to evaporate off, and then tibia were embedded in 100% poly/bed resin and placed in a 60 °C oven for 48 hr. Thin sections (75 nm) were cut using a Reichert-Jung UltraCut E microtome and a Diatome diamond knife. Thin sections were positioned on grids and stained with uranyl acetate and Reynold’s lead citrate. These sections were then imaged on a JEOL JEM-2100 transmission electron microscopy (TEM) (JEOL USA, Inc, Peabody, MA). Representative images are shown at 4000× direct magnification.

### Statistical analysis

**Figure 1:**
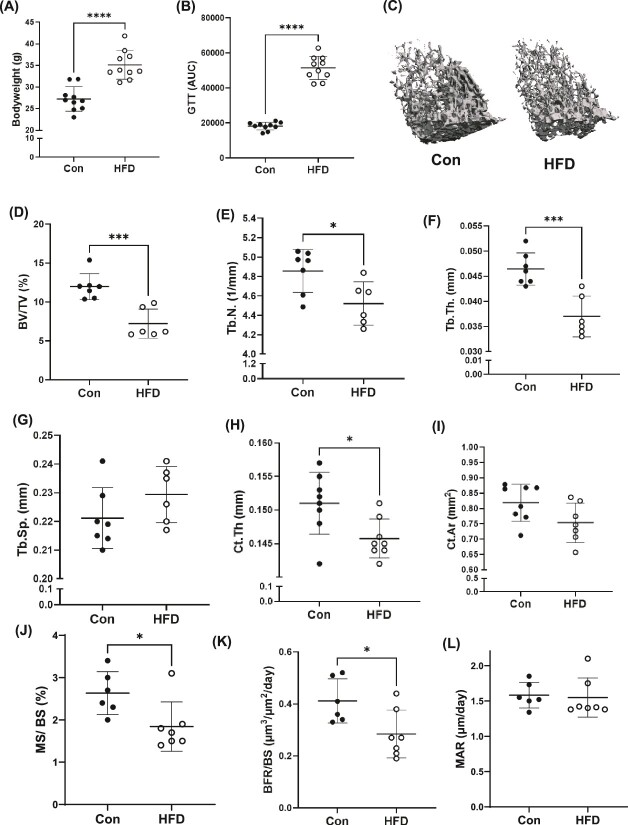
Effect of high fat diet (HFD) on systemic metabolism and skeletal parameters. (A) Body weight and (B) fasting glucose tolerance test of C57BL/6N mice fed a control diet (con) or HFD for 8 wk. (C) Representative 3-D micro-computed tomography images of trabecular bone at the distal femur metaphysis. Trabecular bone parameters include (D) bone volume over total volume (%); (E) trabecular number (Tb.N; mm^−1^); (F) trabecular thickness (Tb.Th; mm); and (G) trabecular separation (Tb.Sp; mm). Each dot represents data from individual animals, where (*n* = 6-7). Cortical bone parameters include (H) thickness (Ct.Th; mm) and (I) area (Ct.Ar; mm^2^). Bone histomorphometry analysis of the proximal tibia to include (J) mineralization surface over bone surface; (K) mineral apposition rate (μm·d^−1^); and (L) bone formation rate over bone surface (μm^3^·μm^−2^·d^−1^). Each dot represents data from individual animals (*n* = 6-10). Statistical comparisons are between the two groups using unpaired *t*-tests. All results are expressed as mean ± SD. ^*^p < .05, ^**^p < .01, ^***^p < .001, ^****^p < .0001.

**Figure 2:**
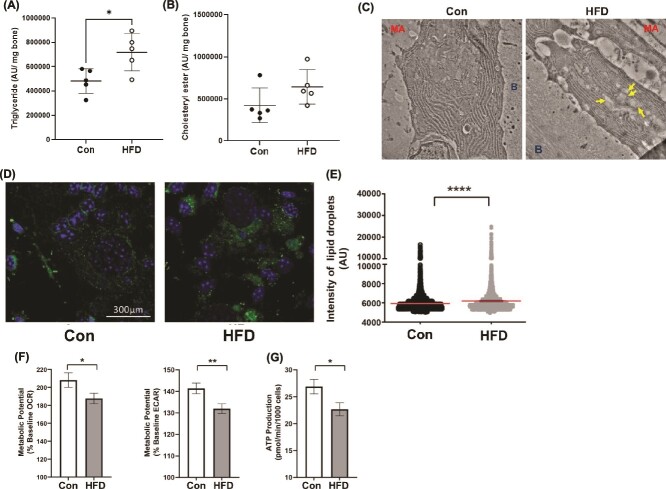
Lipid metabolism in bone and osteoblasts. Thin-layer chromatogram of lipids harvested from flushed tibias following 8 wk on a control (Con) and high fat diet (HFD) of (A) triglyceride and (B) cholesteryl ester normalized to bone weight. Each dot represents data from individual animals (*n* = 5). All results are expressed as mean ± SD. (C) Transmission electron microscopy image of thinly sectioned tibia depicting lipid droplets (yellow arrow) in the cytosol of osteoblasts positioned in between the bone surface (B) and marrow area in control (Con) and HFD fed mice. The images were taken at 4000× magnification. (D) Representative confocal image of ex vivo bone marrow stromal cells following 8 days in osteogenic differentiation medium from control (Con) and high fat diet (HFD) fed mice in which cellular lipid droplets were stained with BODIPY 493/503 and DAPI. Quantification of (E) intensity of BODIPY 493/503-stained lipid droplets in differentiated osteoblasts from control or high fat diet fed mice, where each dot represents the intensity of one lipid droplet. (F) Seahorse cell energy phenotype assay for metabolic potential measured in terms of percentage baseline oxygen consumption rate and percentage extracellular acidification rate and (G) Adenosine triphosphate production rates. Seahorse data are from cells pooled from *n* = 10 animals from each group and seeded with technical replicates for the cell culture experiments. Statistical comparisons are between the two groups using unpaired *t*-tests. ^*^p < .05, ^**^p < .01, ^***^p < .001, ^****^p < .0001.

**Figure 3:**
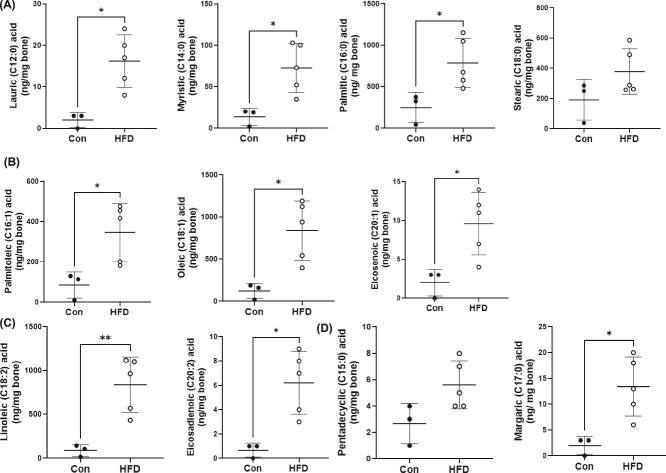
Quantification of long chain fatty acids in the tibia cortex following a control (con) or high fat diet. Mass spectrometric quantification of (A) saturated fatty acids lauric (C12:0), myristic (C14:0), palmitic (C16:0), and stearic acid (C18:0); (B) monounsaturated fatty acids palmitoleic (C16:1), oleic (C18:1), eicosenoic acid (C20:1); (C) Di-unsaturated fatty acids linoleic acid (C18:2) and eicosadienoic acid (C20:2); (D) odd chain numbered fatty acids pentadecylic acid (C15:0) and margaric acid (C17:0). Fatty acid quantity is normalized to bone tissue weight. Each dot represents data from individual animal (*n* = 3-5). All results are expressed as mean ± SD. Statistical comparisons are between the two groups using unpaired *t*-tests. ^*^p < .05, ^**^p < .01, ^***^p < .001, ^****^p < .0001.

**Figure 4:**
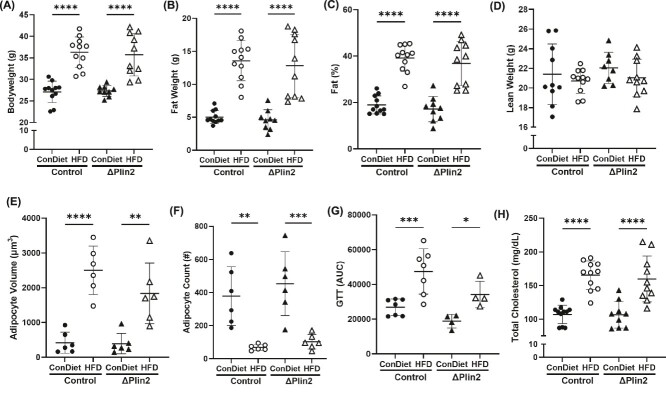
Targeted knockdown of Perilipin2 in osteoprogenitor cells does not affect systemic metabolism. (A) Body weight of control mice or ΔPlin2 mice fed a control (ConDiet) or high fat diet for 12-wk (*p*-value: genotype = 0.0592; diet = <0.0001). (B) Fat mass (*p*-value: genotype ≤ 0.0423; diet ≤ 0.0001), (C) fat percentage (*p*-value: genotype = 0.0828; diet ≤ 0.0001), and (D) lean body mass (*p*-value: genotype = 0.9304; diet = 0.5699). Histological analysis of subcutaneous adipocyte (E) volume (*p*-value: Genotype = 0.6992; diet ≤ 0.0001) and (F) number (*p*-value: genotype = 0.4519; diet = 0.0002) following H&E-staining. (G) Area under the curve following a fasting, intraperitoneal glucose tolerance test (*p*-value: genotype = 0.2204; diet = 0.0010) and (H) serum total cholesterol (*p*-value: genotype = 0.0454; diet ≤ 0.0001). Each dot represents data from individual animal, (*n* = 6-11). All results are expressed as mean ± SD. Significant differences were established using 2-way analyses of variance (2-way ANOVA) with genotype and diet as independent variables, with post-hoc uncorrected Fisher’s LSD tests. Values of *p* < .05 were considered significant, with *p*-values depicted as ^*^p < .05, ^**^p < .01, ^***^p < .001, ^****^p < .0001.

**Figure 5:**
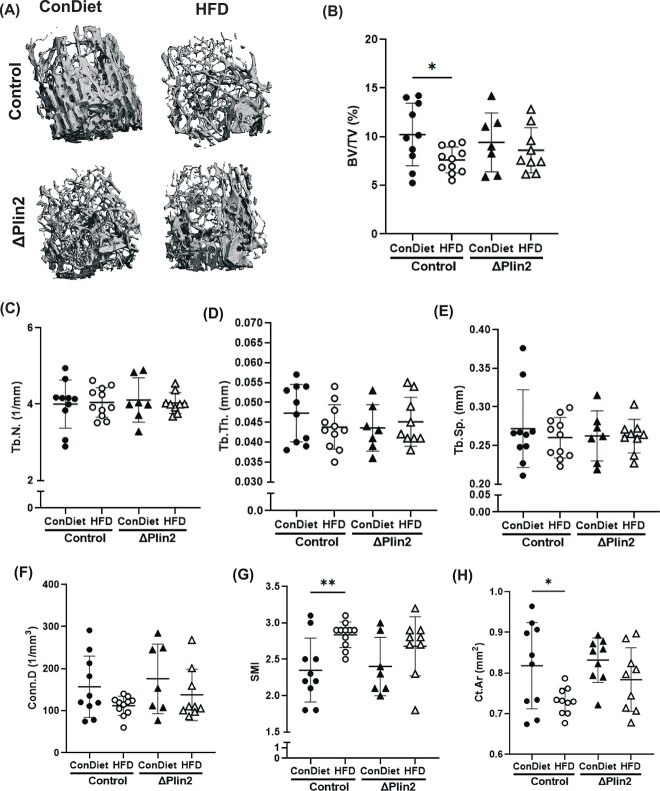
High fat diet-induced compromise in bone microarchitecture is protected in ΔPlin2 mice. (A) Representative 3D micro-computed tomography images of trabecular bone from the distal femur metaphysis. Parameters include (B) bone volume over total volume (%) (*p*-value: genotype = 0.8557; diet = 0.0503); (C) trabecular (Tb.N; mm^−1^) (*p*-value: genotype = 0.0752; diet = 0.6424); (D) trabecular thickness (Tb.Th; mm) (*p*-value: genotype = 0.5286; diet = 0.5618); (E) trabecular separation (Tb.Sp; mm) (*p*-value: genotype = 0.1811; diet = 0.7020); (F) connectivity density (Conn.D; 1/mm^3^) (*p*-value: genotype = 0.4503; diet = 0.0996); (G) structural model index (*p*-value: genotype = 0.7465; diet = 0.0272). Cortical bone analysis of the femur mid-diaphysis includes (H) cortical bone area (Ct.Ar; mm^2^) (*p*-value: genotype = 0.3800; diet = 0.0240). Each dot represents data from individual animal (*n* = 7-10). All results are expressed as mean ± SD. Significant differences were established using 2-way analyses of variance (2-way ANOVA) with genotype and diet as independent variables, with post-hoc uncorrected Fisher’s LSD tests. Values of *p* < .05 were considered significant, with *p*-values depicted as ^*^p < .05, ^**^p < .01, ^***^p < .001, ^****^p < .0001.

**Figure 6:**
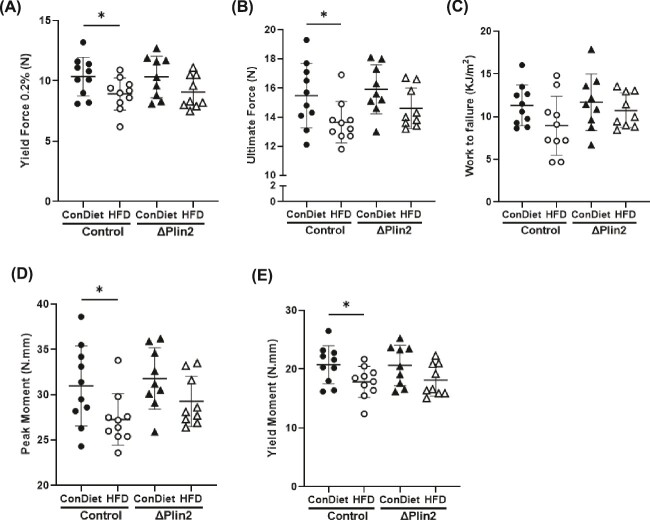
ΔPlin2 mice display protection against high-fat diet (HFD)-induced bone fragility. Three-point bending analysis of the femur from control diet (ConDiet) or HFD fed control or ΔPlin2 mice. Parameters include (A) yield force (N) (*p*-value: genotype = 0.5299; diet = 0.0809); (B) ultimate force (N) (*p*-value: genotype = 0.5748; diet = 0.0409); (C) work to failure (KJ/m^2^) (*p*-value: genotype = 0.5157; diet = 0.2230); (D) peak moment (N.Mm) (*p*-value: genotype = 0.5807; diet = 0.0415); and (E) yield moment (N.Mm) (*p*-value: genotype = 0.521; diet = 0.0801). Each dot represents data from individual animal (*n* = 9-10). All results are expressed as mean ± SD. Significant differences were established using 2-way analyses of variance (2-way ANOVA) with genotype and diet as independent variables, with post-hoc uncorrected Fisher’s LSD tests. Values of *p* < .05 were considered significant, with *p*-values depicted as ^*^p < .05, ^**^p < .01, ^***^p < .001, ^****^p < .0001.

Statistical analyses were carried out using GraphPad Prism 10.3. Biological *n*’s are indicated in each corresponding figure legend. For all comparisons between two groups (Figures 1-3), significant differences were established using unpaired *t*-tests. For all comparisons with four groups (Figures 4-6) significant differences were established using 2-way analyses of variance (2-way ANOVA) with genotype and diet as independent variables, with post-hoc uncorrected Fisher’s least significant difference (LSD) tests. Values of *p* < .05 were considered significant, with *p*-values depicted as ^*^p < .05, ^**^p < .01, ^***^p < .001, ^****^p < .0001. All results are expressed as mean ± SD.

## Results

### Diet induced obese mouse model recapitulated T2DM phenotype while impairing osteoblast function and bone formation

As confirmation of our model, mice on the HFD, weighed significantly more compared to mice fed the control diet (Con) diet for 8 wk (Figure 1A). The mice on the HFD demonstrated glucose intolerance as evidenced by glucose tolerance tests (GTT) AUC (Figure 1B). Further, in line with previous reports, our data also showed reduced trabecular bone parameters in mice fed a HFD compared to controls (Figure 1C). Trabecular bone of the distal femur metaphysis from mice fed a HFD for 8 wk demonstrated significantly lower bone volume (BV)/total volume (TV) (Figure 1D), trabecular number (Figure 1E), and trabecular thickness (Figure 1F), with no significant changes detected in trabecular separation (Figure 1G). Trabecular connectivity density was also lower in the mice fed the HFD compared to the control mice (Figure S1A). Structural model index (SMI) also indicated a “weaker” bone with rod-like trabecular structures from mice on the HFD compared to the plate-like struts in the control diet fed mice (Figure S1B). Cortical bone thickness was also significantly reduced in mice fed the HFD compared to controls (Figure 1H), while no change was detected in cortical area (Figure 1I). No changes were noted in cortical bone medullary volume or moment of inertia between dietary treatments (Figure S1C). There was also no change in the length of either femur or tibia on the HFD compared to Con diet (*data not shown*). Bone histomorphometry analysis revealed reduced osteoblast activity in mice fed the HFD quantified by lower mineralization surface per bone surface (MS/BS) as well as reduced bone formation rates (BFR/BS) compared to mice on Con diet; however, no changes were detected in mineral apposition rate (Figure 1J-L).

### High-fat diet resulted in impaired lipid metabolism in bone cortex

Total lipids harvested from the tibia cortex demonstrated an altered lipid profile as triglycerides were significantly elevated in mice fed the HFD compared to Con diet group (1.5-fold) (Figure 2A). Another neutral lipid, cholesteryl ester, showed a trend toward an increase in HFD fed mice, but this was not statistically significant (Figure 2B). We next performed TEM to image osteoblasts located on the endocortical surface of the tibia such that bone marrow can be observed on one side of the osteoblast, while bone is detected on the other. These images revealed that while mice fed the Con diet maintained healthy, plump, cuboidal osteoblasts with well-defined endoplasmic reticulum, osteoblasts from the HFD fed mice had lipid droplets accumulating withing their cytoplasm (Figure 2C). Consistent with these data, BMSCs isolated from mice fed the HFD when cultured in osteogenic medium exhibited an increase in neutral lipid droplets compared to control diet (Figure 2D). These lipid droplets were further quantified as increased intensity of BODIPY 493/503 in osteoblasts from HFD mice compared to those from Con diet fed mice (Figure 2E). Finally, these ex vivo BMSC-derived osteoblast cultures demonstrated that mice fed a HFD had impaired metabolic potential in respect to both baseline OCR and ECAR (Figure 2F). This metabolic dysfunction in BMSC-derived osteoblasts cultured from mice on a HFD produced less ATP compared to mice fed Con diet (Figure 2G). These data demonstrate that osteoblasts from mice fed a HFD display lipid dysfunction along with impaired bioenergetic capacity.

Since we were unable to confidently quantify fatty acids using TLC (Figure S2), lipids harvested from the tibia cortex were analyzed for LCFAs using mass spectrometry. These data revealed a higher abundance of three saturated fatty acids, lauric acid (C12:0), myristic acid (C14:0), and palmitic acid (C16:0) during HFD feeding compared to that in Con-diet fed mice (Figure 3A). Stearic acid (C18:0) also showed a trend toward an increase in HFD fed group, although it did not reach statistical significance (Figure 3A). Monounsaturated fatty acids, palmitoleic acid (C16:1), oleic acid (C18:1), and eicosenoic acid (C20:1), as well as unsaturated fatty acids, linoleic acid (C18:2) and eicosadienoic acid (C20:2), were also present in higher amounts in the HFD fed mice compared to that of Con diet fed mice respectively (Figure 3B and C). Interestingly, we detected the presence of two odd chain saturated fatty acids pentadecylic acid (C15:0) and margaric acid (C17:0), where margaric acid was also significantly increased in the tibia cortex in mice fed a HFD (Figure 3D).

### Knocking down *Plin2* in osteoblast progenitor cells in mice had no effect on high-fat diet induced alterations in central metabolism

Prx1^Cre/+;^Plin2^fl/lfl^, hereafter referred to as “ΔPlin2”, and Cre negative littermate controls (Prx1^+/+^;Plin2^fl/lfl^), hereafter referred to as “Control”, were generated as described in the methods section. Both male and female ΔPlin2 mice were born as per the expected Mendelian frequency. The osteoprogenitor specific ΔPlin2 mice exhibited 6.4-fold reductions in Plin2 protein expression in the bone cortex (devoid of marrow) (Figure S3A). Ex vivo differentiation of osteoblasts derived from BMSCs also showed a 1.4-fold reduction in the expression of *Plin2* mRNA (Figure S3B). As for the male mice, regardless of genotype, both control and ΔPlin2 mice on the HFD demonstrated expected increase in body weight, whole body fat mass, and body fat percentage compared to mice on the Con diet (Figure 4A-C). No changes were detected in lean mass (Figure 4D). HFD-induced adipocyte hypertrophy was noted in subcutaneous adipose depots as evidenced by an increase in adipocyte volume (Figure 4E) and a decrease in adipocyte number (Figure 4F) compared to Con diet. Mice from both genotypes, control and ΔPlin2, demonstrated glucose intolerance as indicated by increased GTT AUC in response to the HFD compared to Con diet (Figure 4G). A cohort of mice were singling housed for 4 days to collect food intake data, which was not significantly different between genotypes. Control mice on the Con diet consumed 1.89 ± 0.27 g·d^−1^ (7.27 ± 1.04 kcal·d^−1^) and 1.99 ± 0.81 g·d^−1^ (10.47 ± 4.3 kcal·d^−1^) of the HFD. This was similar to the ΔPlin2 mice which consumed 2.06 ± 0.55 g·d^−1^ (7.92 ± 2.13 kcal·d^−1^) of the Con diet and 1.74 ± 0.57 g·d^−1^ (9.13 ± 2.98 kcal·d^−1^). Dyslipidemia was further confirmed by an increase in total serum cholesterol in both control and ΔPlin2 mice fed a HFD (Figure 4H). Consistent with these changes there was an increase in serum HDL in mice fed the HFD compared to Con diet (Figure S4A). Interestingly, serum triglycerides were elevated in ΔPlin2 mice fed the HFD compared to the control mice fed the HFD (Figure S4B). No changes were observed in serum non-esterified fatty acids (NEFA) in response to genotype or diet (Figure S4C). The male responsiveness to the HFD was more robust, however, female mice on the HFD did display significant fat weight gain, impaired glucose tolerance, and hypercholesterolemia compared to mice fed Con diet in both genotypes (Figure S5A-E). Taken together these data demonstrate that ΔPlin2 mice responded similarly to control mice fed a HFD as they experienced an increase in adiposity, demonstrated dyslipidemia, and developed glucose intolerance.

### Loss of function of Plin2 in osteoblast progenitor cells provided protection from high-fat diet induced low bone mass phenotype by increasing bone formation

Knocking out Plin2 in osteoblast progenitor cells (ΔPlin2) protected from the HFD-associated decrease in trabecular BV, which, as expected, was observed in control male mice on the HFD (Figure 5A and B). Despite these changes in BV/TV, there were no significant differences in trabecular number, thickness, or separation (Figure 5C-E). No changes were noted in connectivity density regardless of diet or genotype (Figure 5F). SMI was indicative of “weaker bone” or more rod-like trabeculae in control mice fed a HFD when compared to Con diet, whereas in ΔPlin2 mice there was no change in SMI between HFD and Condiet (Figure 5G). Analysis of the femur diaphysis demonstrated that ΔPlin2 mice were protected from HFD-mediated reduction in cortical area (Figure 5H) and moment of inertia (MMOI) (Figure S6A). No change was detected in cortical thickness between dietary or genotype (Figure S6B). As expected, given our targeted deletion of Plin2 using the Prrx1-Cre, no changes were detected in microarchitectural parameters of the vertebra between genotypes or dietary treatment (Figure S6C). Interestingly, despite female mice responding to the HFD with increased adiposity and impaired glucose tolerance, there were no changes in trabecular BV/TV, number, or thickness in response to the HFD in female mice (Figure S7A-C). There was an increase in trabecular separation in the HFD-fed female control mice compared to those fed the Con diet (Figure S7D). This was consistent with no changes detected in vertebral BV/TV of female mice with diet or genotype (Figure S7E). Given the lack of skeletal changes within the female mice in response to the HFD, for all further analyses only male mice were used.

Bone histomorphometry analysis of the proximal tibia supported that the reduction observed in control mice fed a HFD was a result of impaired osteoblast activity indicated by lower mineralization apposition rate (MAR) as well as reduced bone formation rates (BFR/BS) (Table 1). Consistent with our microarchitectural parameters, these indices were protected, and similar between Con and HFD-fed ΔPlin2 mice (Table 1). Control and ΔPlin2 mice fed a HFD also resulted in an increase in bone marrow adiposity compared to mice fed a Con diet (Table 1). There were no changes in the number of osteoblasts (N.Ob/BS) or osteoclasts on the bone surface (N.Oc/BS) in either of the genotypes (Table 1).

**Table 1:** Dynamic and static bone histomorphometry of the proximal tibia metaphysis.

	Control		ΔPlin2	
	ConDiet	HFD	ConDiet	HFD
**MS/BS (%)**	2.4 ± 0.7	1.8 ± 0.8	2.1 ± 0.8	1.9 ± 1.1
**MAR (μm·d^−1^)**	2.035 ± 0.45	1.59 ± 0.23^*^	1.57 ± 0.41	1.51 ± 0.42
**BFR/BS (μm^3^·μm^−2^·d^−1^)**	0.489 ± 0.17	0.302 ± 0.16^*^	0.331 ± 0.16	0.30 ± 0.19
**N.Ob/BS (N/mm)**	44.50 ± 11.8	38.26 ± 13.9	49.66 ± 18.9	41.33 ± 13.6
**BMAd.V/TV (%)**	0.67 ± 0.97	6.46 ± 5.78^**^	1.11 ± 1.33	4.89 ± 3.44^*^
**N.Oc/BS (N/mm)**	5.51 ± 3.2	5.07 ± 2.9	10.18 ± 3.77	6.82 ± 0.85

Control mice and ΔPlin2 mice were fed a control (ConDiet) or high-fat diet (HFD) for 12 wk. Parameters shown are mineralization surface per bone surface (MS/BS) (*p*-value: genotype = 0.3947; diet = 0.6049); mineral apposition rate (MAR; μm·d^−1^) (*p*-value: genotype = 0.7471; diet = 0.0541); bone formation rate over bone surface (BFR/BS; μm^3^·μm^−2^·d^−1^) (*p*-value: genotype = 0.4669; diet = 0.1202); number of osteoblasts per bone surface (N.Ob/BS; Number per mm) (*p*-value: genotype = 0.8772; diet = 0.4382); bone marrow adipocyte volume per total volume (BMAd.V/TV; %) (*p*-value: genotype = 0.8878; diet = 0.0057); number of osteoclasts per bone surface (N.Oc/BS; Number per mm) (*p*-value: genotype = 0.8204; diet = 0.3287). All the data are presented as the mean ± SD (*n* = 4-10). Statistical comparisons were made using 2-way analyses of variance (2-way ANOVA) with genotype and diet as independent variables, with post-hoc uncorrected Fisher’s LSD tests. Values of *p* < .05 were considered significant, with *p*-values depicted as ^*^p < .05, ^**^p < .01, ^***^p < .001, ^****^p < .0001.

### Knocking out Plin2 in osteoblast progenitor cells protected bone from high-fat diet induced skeletal fragility

Biomechanical testing was performed on femur specimens and demonstrated that HFD in control mice led to decreased yield force (Figure 6A) and ultimate force (Figure 6B); however, these parameters were protected from this HFD-induced decrease in bone quality in the ΔPlin2 mice. No change was detected in work to failure regardless of genotype or diet (Figure 6C). Furthermore, both peak and yield moment were decreased in control mice on the HFD, without any alterations in the ΔPlin2 mice (Figure 6D and E).

### Absence of Plin2 in osteoblast progenitor cells increased fatty acid transfer to mitochondria for β-oxidation

**Figure 7:**
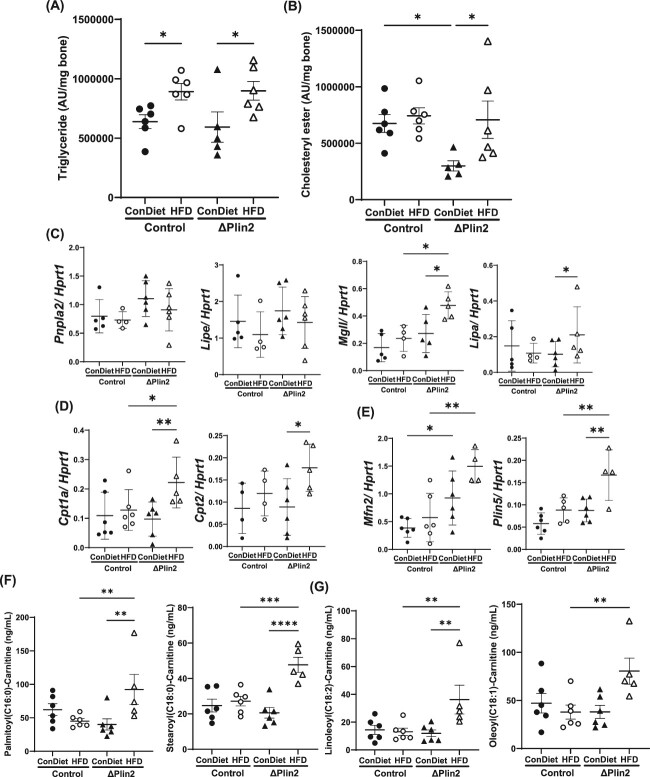
Targeted knockdown of Plin2 in osteoprogenitor cells alters lipid metabolism in bone. Densitometric quantification from thin layer chromatography (TLC) of the lipid species harvested from flushed tibias (A) triglyceride (*p*-value: genotype = 0.4646; diet = 0.0373); (B) cholesteryl ester (*p*-value: genotype = 0.3773; diet = 0.0433) normalized to bone weight. Quantitative real time PCR of target genes involved in (C) lipolysis [Pnpla2 (*p*-value: genotype = 0.3006; diet = 0.2900), Lipe (*p*-value: genotype = 0.0258; diet = 0.4887), Mgll (*p*-value: genotype = 0.8772; diet = 0.0139), Lipa (*p*-value: genotype = 0.0515; diet = 0.1669)]; (D) fatty acid oxidation [Cpt1a (*p*-value: genotype = 0.2261; diet = 0.0429), Cpt2 (*p*-value: genotype = 0.7165; diet = 0.0987)]; and (E) mitochondria-lipid droplet contact sites [Mfn2 (*p*-value: genotype = 0.6696; diet = 0.0071), Plin5 (*p*-value: genotype = 0.2280; diet = 0.0009)] normalized to Hprt1 from flushed femurs. Mass spectrometric quantification of carnitinylated long chain fatty acids within the tibia cortex to include (F) carnitinylated saturated fatty acids palmitoyl (C16:0)-carnitine (*p*-value: genotype = 0.1689; diet = 0.0165), stearoyl (C18:0)-carnitine (*p*-value: genotype = 0.3166; diet = 0.0002); (G) unsaturated fatty acids linoleoyl (C18:2)-carnitine (*p*-value: genotype = 0.4155; diet = 0.0139), and oleoyl (C18:1)-carnitine (*p*-value: genotype = 0.2562; diet = 0.0106). Each dot represents data from individual animal. All results are expressed as mean ± SD. Significant differences were established using 2-way analyses of variance (2-way ANOVA) with genotype and diet as independent variables, with post-hoc uncorrected Fisher’s LSD tests. Values of *p* < .05 were considered significant, with *p*-values depicted as ^*^p < .05, ^**^p < .01, ^***^p < .001, ^****^p < .0001.

To further characterize lipid handling changes occurring in vivo we performed crude lipid profiling of the tibia cortex, which did not include growth plates or marrow elements. Both control and ΔPlin2 mice fed a HFD displayed increased triglycerides in the bony cortex compared to control diet (Figure 7A). Interestingly, cholesteryl ester was significantly decreased in ΔPlin2 mice compared to control mice on the Con diet, but HFD only increased cholesteryl esters in the ΔPlin2 mice (Figure 7B) between either group. Confocal imaging further demonstrated a decrease in cytosolic lipid droplets from ex vivo differentiated osteoblasts (day 8 of differentiation) only in the ΔPlin2 mice (Figure S8A). This was quantified by an increase in intensity (4624 AU ± 23.45) and number (165.3 ± 33.81) compared to the lipid droplets in cells cultured from control mice (4128 AU ± 21.43 intensity and 54.59 ± 5.80) (Figure S8B and C, respectively). These data further indicated the knockout of Plin2 was exerting a functional effect on lipid metabolism within osteoblasts.

Gene expression profiling of the bone cortex devoid of marrow elements demonstrated no changes in lipases including *Pnpla2* or *Lipe*. However, *Mgll* and *Lipa* were up-regulated in the ΔPlin2 mice fed the HFD compared to Con diet (Figure 7C). Further, expression of *Cpt1* and *Cpt2,* genes coding for the rate limiting enzymes in fatty acid β-oxidation, were upregulated in ΔPlin2 mice fed a HFD (Figure 7D). Finally, two mitochondrial-lipid droplet association protein coding genes, *Mfn2* and *Plin5,* were also upregulated in ΔPlin2 mice fed a HFD (1.8-fold and 1.9-fold, respectively; Figure 7E). These data support the role of Plin2 in regulating lipid metabolism by mechanisms related to lipolysis, lipophagy and/or cholesterol handling, and mitochondrial-lipid droplet contact sites.

Given the complexity in studying a dynamic process such as lipid flux, we next used novel mass spectrometry to detect carnitinylation of fatty acids as an indicator of β-oxidation as LCFAs must first be transported into the mitochondria via carnitine pathways. Consistent with our gene expression data and genetic deletion, analysis of the bone cortex showed that ΔPlin2 mice on the HFD had an increase in carnitylation of saturated fatty acids including palmitoyl (C16:0)- and stearoyl (C18:0)-carnitine (Figure 7F). This increase in carnitine fatty acids within the ΔPlin2 mice on the HFD was also demonstrated in the unsaturated fatty acid, linoleoyl (C18:2) (Figure 7G), compared to ΔPlin2 on the Con diet. Oleoyl-carnitine was also significantly higher in the ΔPlin2 mice fed the HFD when compared to control mice on the HFD diet (Figure 7G). These data are the first of their kind; however, we recognize the limitations of the sample isolation. For example, the tibia cortex was devoid of marrow elements and growth plate, therefore we expect this profile to reflect the enriched populations of osteoblasts and osteocytes. However, it’s also possible some vascular endothelial cells are contaminating these samples. Moreover, within the bone itself, we are unable to differentiate osteoprogenitors and/ or osteoblasts on the endocortical surface vs periosteum but are actively working to delineate such spatial distribution.

## Discussion

The current study demonstrates the importance of osteoblast lipid metabolism during HFD related metabolic perturbations such as dyslipidemia, hyperglycemia, and impaired glucose tolerance. It has been appreciated that lipid dysfunction contributes to many pathologies associated with obesity-related conditions, such as T2DM, including hepatic steatosis and atherosclerosis.^14^^,^^36^ This study further expands on this to include osteoblast lipid dysfunction as a contributing factor resulting in reduced bone formation and subsequent reduction in bone strength, which is also observed in patients with T2DM.^37^ The current study also demonstrates the ability to selectively increase lipolysis in osteoblast-progenitor cells by genetic deletion of Plin2, which protects from HFD-related deleterious effects on the skeleton. We recognize the complexities of the increased bone fragility in T2DM,^37^ but our findings for the first time highlight that osteoblast lipid metabolism is altered during related conditions and is likely a contributing factor for increased fragility observed in patients with T2DM.

Our data further establish that chronic HFD feeding manifests in weight gain and impaired glucose tolerance, but also results in lipid droplet accumulation in osteoblasts, both in vivo and ex vivo. Given the dynamic nature of lipid droplet flux, including lipid droplet biogenesis and breakdown via lipolysis, two possible scenarios exist.^38^ The first, is that exogenous dietary derived fatty acids are “flooding” the osteoblast such that chylomicrons delivering fatty acids to osteoblasts are being transported intracellularly and esterified rapidly to form the triacylgerol within the lipid droplet core. The second, is that lipid droplets have already formed, and their lipolysis is impaired, again resulting in greater number of lipid droplets. Based on these data, both scenarios likely contribute to increased lipid droplet and lipid content within the osteoblasts. However, we were struck by the reduced osteoblast metabolic potential and ATP production during HFD feeding. Our lab has recently expanded on the functional role of lipid droplets within osteoblasts to serve as a fatty acid reservoir, mobilized when bioenergetic capacity, or ATP production, needs to be maintained.^17^ We established that when lipolysis is blocked in osteoblasts, it results in impaired osteoblast function, reduced bone formation rates, and increased osteoblast lipid accumulation.^17^ Hence, our previous model of impaired lipolysis^17^ mirrors the lipid disturbances observed in the diet induced obesity mouse model of T2DM.

To further expand on this point, while HFD feeding resulted in the accumulation of fatty acids within the bone cortex, given their impaired metabolic profile, these fatty acids were likely not contributing to bioenergetic capacity, and in that regard could be cytotoxic. For example, just because cells accumulate substrates, fatty acids in this case, do not necessarily translate to sufficient catabolism for ATP production. Therefore, while our data and others demonstrate the importance of fatty acids for osteoblast function and skeletal health, specifically LCFAs, for mitochondrial β-oxidation,^39–41^ when these fatty acids are not properly stored or oxidized, their intracellular accumulation can be detrimental. While the finely tuned metabolic balance remains unknown within osteoblasts, this study expands on this phenomenon. Furthermore, while the American Institute of Nutrition (AIN) established a rigorous rodent dietary formula to include soybean oil as the primary source of fats, as it supplies appropriate proportion of essential fatty acids linoleic and linolenic acids, commercially available “high fat diets” use other/additional sources. The diet used in the current study, and arguably most frequently, includes lard in addition to soybean oil.^42^^,^^43^ Interestingly, the fatty acid profile detected in the bone cortex, reflects this diet as lard is high in LCFAs including myristate, palmitate, stearate, and oleate.^26^ Interestingly, odd chain saturated fatty acids pentadecanoic acid [C15:0] and heptadecanoic acid [C17:0] were also elevated in the bone cortex and have been documented to be cardioprotective by attenuating inflammation.^44^ This profile demonstrates increased saturated, unsaturated, and polyunsaturated fatty acids within the bone cortex following HFD. While we expect this model to translate to obesity associated metabolic perturbations such as T2DM given the similar pathologies, we recognize the dietary composition is also vastly different among humans and mice. Recently, fatty acid composition was demonstrated to directly influence bone health and osteoblast activity.^45^ Importantly, while dietary fatty acid profiles differed, both led to increased body weight gain, but glucose tolerance was not reported.^45^ Therefore, in future studies, it will be interesting to dissect out the influence of dietary composition vs metabolic disruption as it relates to bone health.

Given these data supporting the idea that HFD disrupts osteoblast lipid metabolism contributing to osteoblast dysfunction, coupled with previous studies demonstrating that increasing lipolysis in a tissue specific manner can protect from HFD related pathologies,^22^^,^^23^ we explored whether a similar mechanism could be beneficial in bone. To this end, we generated osteoprogenitor-specific Plin2 conditional knock-down mice to enhance lipolysis and fed them a control diet or HFD. Indeed, our data support our hypothesis that increasing lipolysis in osteoblast-progenitor cells and cells of the osteoblast lineage protect from HFD induced skeletal dysfunction. Osteoblast function was maintained despite HFD feeding in the ΔPlin2 male mice. This resulted in maintained bone microarchitecture and biomechanical properties in the ΔPlin2 mice fed the HFD. We did not detect this same protection in female mice, however, given the evidence that female metabolic programming is different than males, coupled with the fact that the HFD model is inconsistent in female mice, this was not entirely surprising.^17^^,^^21^ Further investigation of the molecular mechanism of altered lipid metabolism in the male mice demonstrated increased lipolysis and β-oxidation in the ΔPlin2 mice on the HFD as indicated by significant increases in *Mgll*, *Cpt1*, and *Cpt2*. While we again demonstrated that HFD increases triglycerides within the bone cortex, there was no significant difference in triglycerides between the ΔPlin2 mice on the control diet vs HFD. While this was somewhat surprising as we expected reduced triglycerides within the bone cortex from ΔPlin2 mice on the HFD, this is likely due to complexities of lipid flux as a dynamic process while our measurements were static. Therefore, we characterized the carnitylated LCFAs within the bone cortex, to quantify fatty acids being transferred into the mitochondria, presumably to undergo β-oxidation. These data demonstrate that despite increased FFAs detected in the bone cortex during HFD in wildtype mice, as expected based on bioenergetic profile, these fatty acids are not undergoing β-oxidation. Conversely, enhancing lipolysis in osteoblast progenitor cells, as in the ΔPlin2 mice, on a HFD results in increased carnitylation of fatty acids, consistent with maintained β-oxidation. Based on these data, ATP production is expected to be conserved, thereby positively regulating osteoblast bioenergetic status protecting cellular function even during HFD metabolic dysfunction.

In summary, these data establish a mechanism whereby HFD induced metabolic perturbations, like those observed in patients with T2DM, results in increased skeletal fragility due to impaired osteoblast lipid metabolism and reduced bioenergetic capacity. Protecting osteoprogenitor cells from impaired lipid metabolism by enhancing lipolysis and fatty acid oxidation via Plin2 knockdown resulted in maintained skeletal health like that observed in control diet fed mice. The precise molecular mechanism(s) that contribute to the lipid dysfunction within the osteoprogenitor cells remains unclear, however it could be a result of toxic FFAs, lipid membrane alterations, mitochondrial dysfunction, and/or bioenergetic capacity. Further studies should elaborate on these mechanisms as it is likely that lipid metabolism impacts osteoblast function and skeletal health in a multifaceted manner.

## Supplementary Material

Supp_Fig_1_zjae195

Supp_Fig_2_zjae195

Supp_Fig_3_zjae195

Supp_Fig_4_zjae195

Supp_Fig_5_zjae195

Supp_Fig_6_zjae195

Supp_Fig_7_zjae195

Supp_Fig_8_zjae195

Supp_Table_1_zjae195

Supp_Table_2_zjae195

Supp_Table_3_zjae195

Supp_Figure_Legends_Clean_zjae195

## Data Availability

Data available on request.
