# Supplementary figures and images for: Enhanced fatty acid oxidation in osteoprogenitor cells provides protection from high-fat diet induced bone dysfunction
Source: J Bone Miner Res. 2024 Dec 8;40(2):283–98. doi: 10.1093/jbmr/zjae195 (PMC11789392; doi:10.1093/jbmr/zjae195)

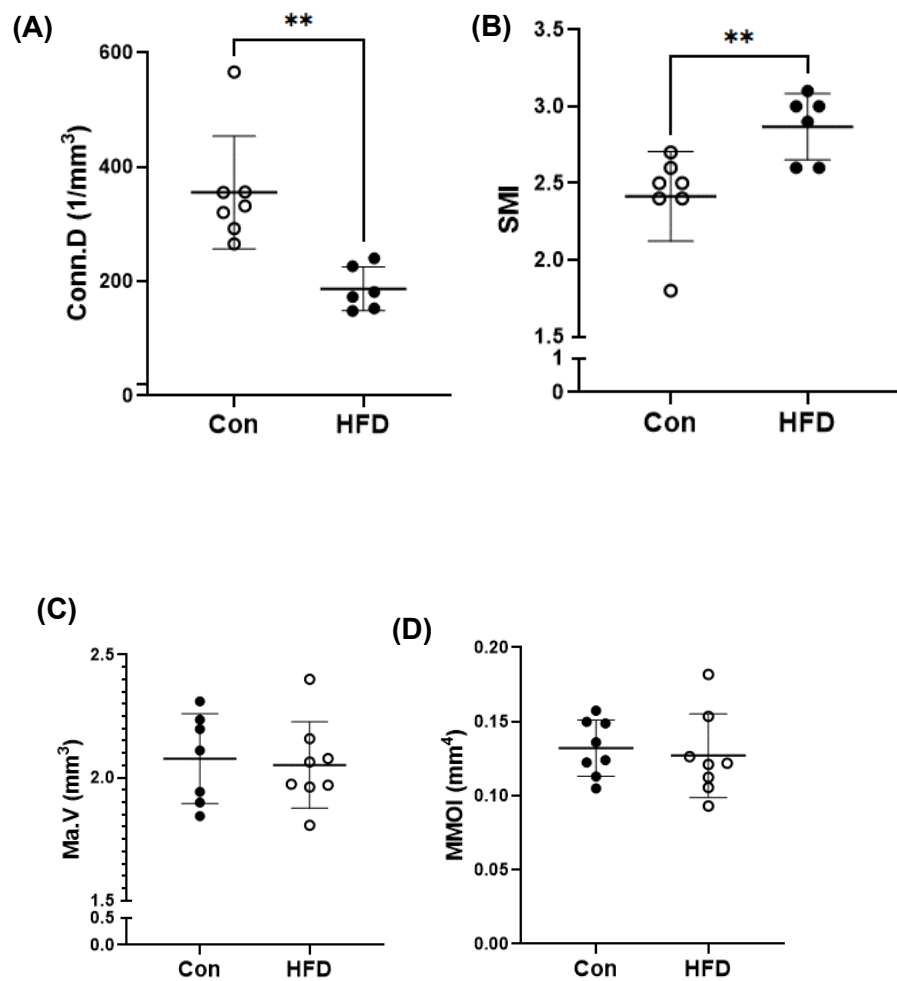

Supplement: Supp_Fig_1_zjae195 [file supp_fig_1_zjae195.pdf]

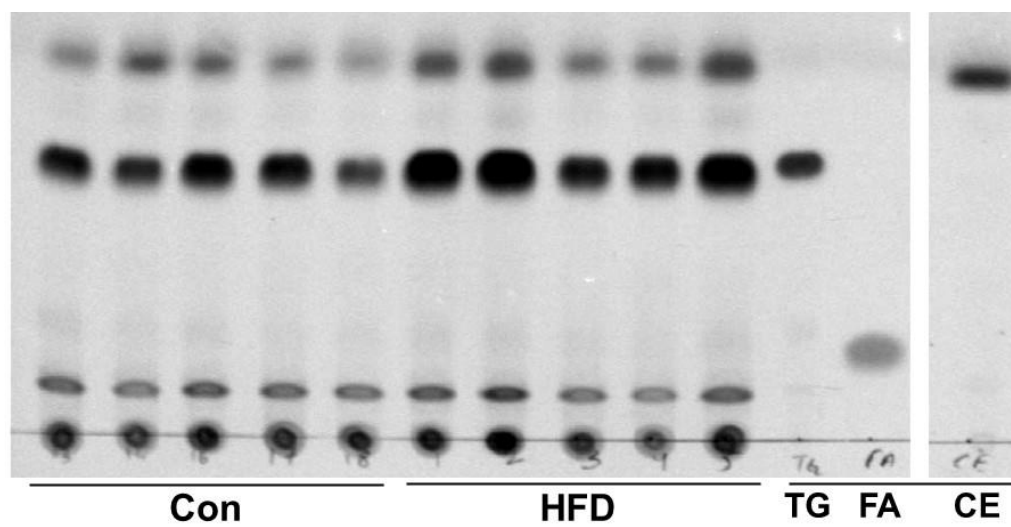

Supplement: Supp_Fig_2_zjae195 [file supp_fig_2_zjae195.pdf]

(A)

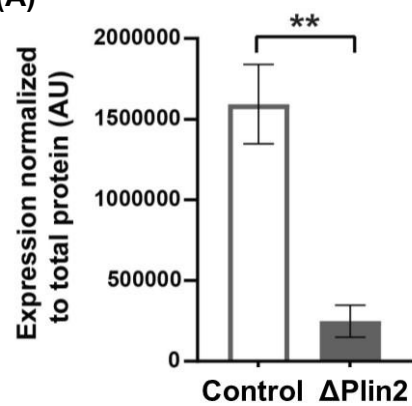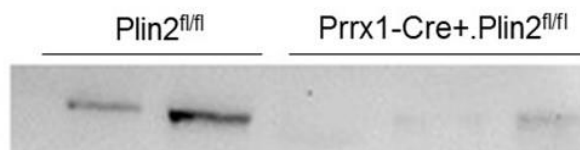

(B)

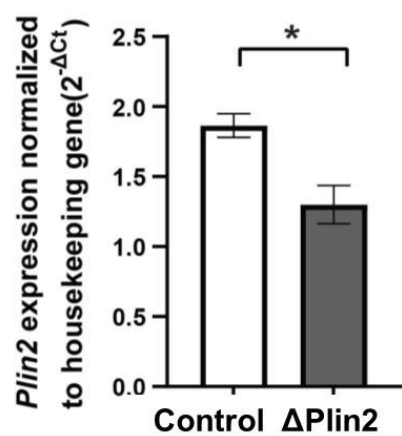

Supplement: Supp_Fig_3_zjae195 [file supp_fig_3_zjae195.pdf]

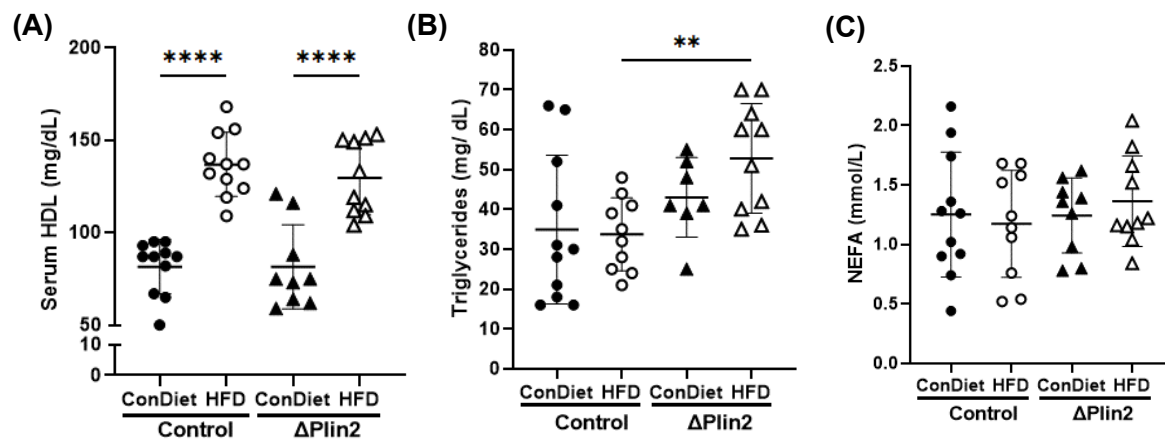

Supplement: Supp_Fig_4_zjae195 [file supp_fig_4_zjae195.pdf]

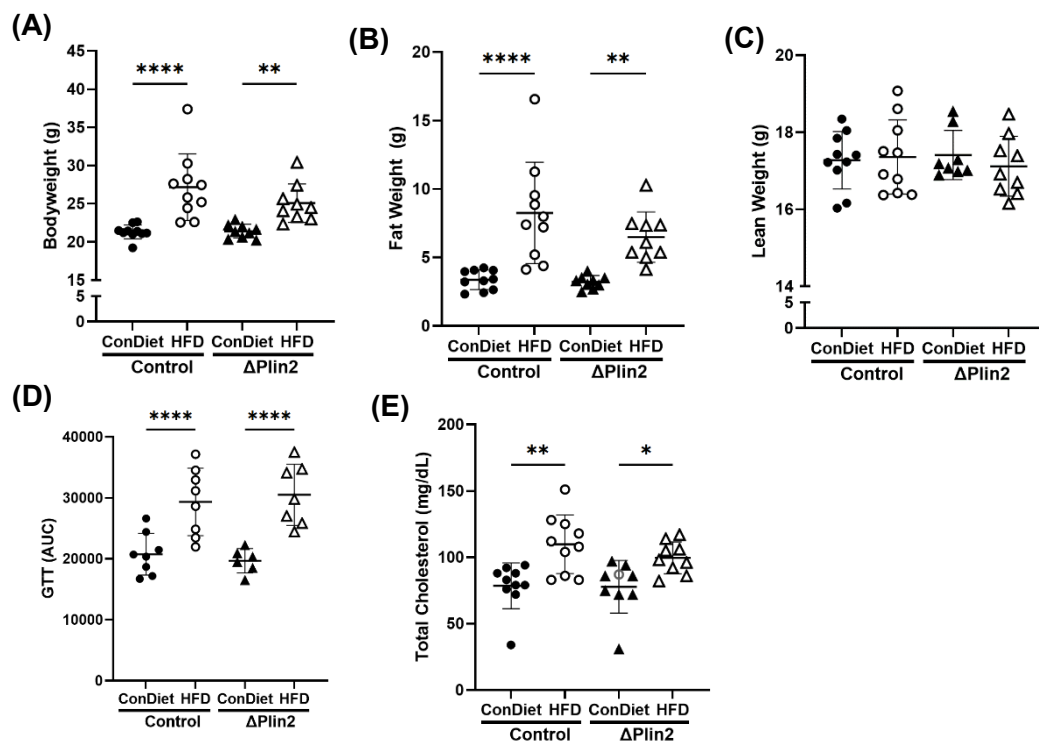

Supplement: Supp_Fig_5_zjae195 [file supp_fig_5_zjae195.pdf]

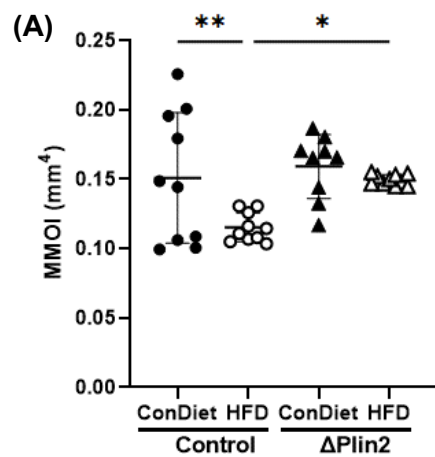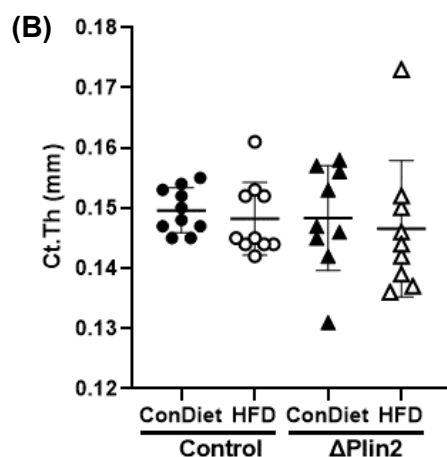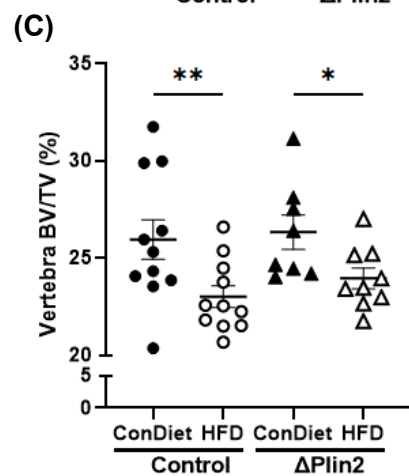

Supplement: Supp_Fig_6_zjae195 [file supp_fig_6_zjae195.pdf]

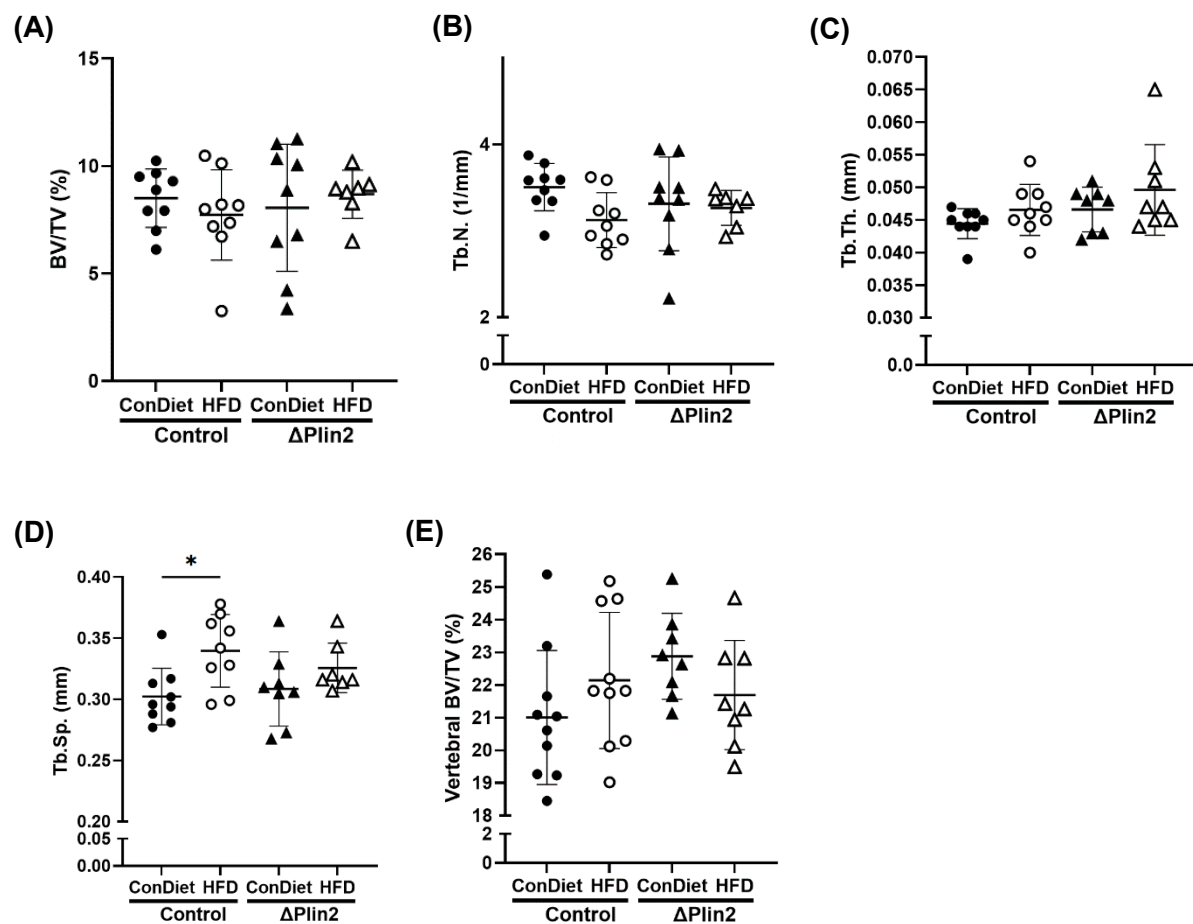

Supplement: Supp_Fig_7_zjae195 [file supp_fig_7_zjae195.pdf]

**(A)**

Control

## $\Delta$ Plin2

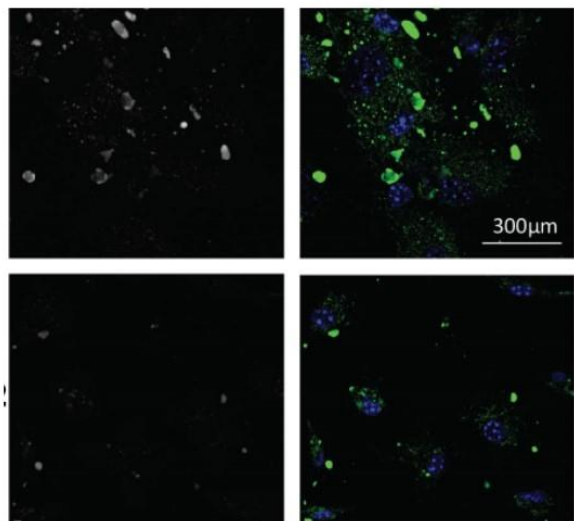

**(B)**

**Intensity of Lipid droplets(AU)**

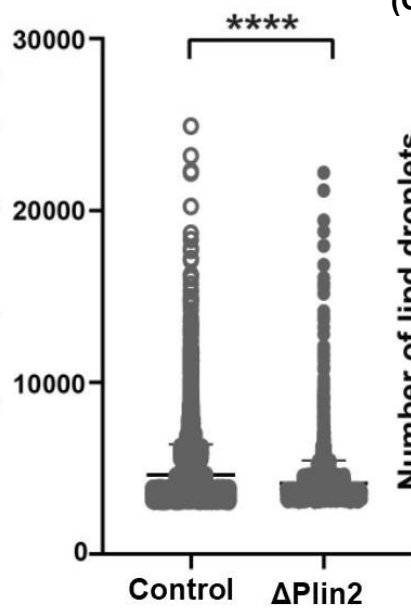

(C)

**Number of lipid droplets  
per cell**

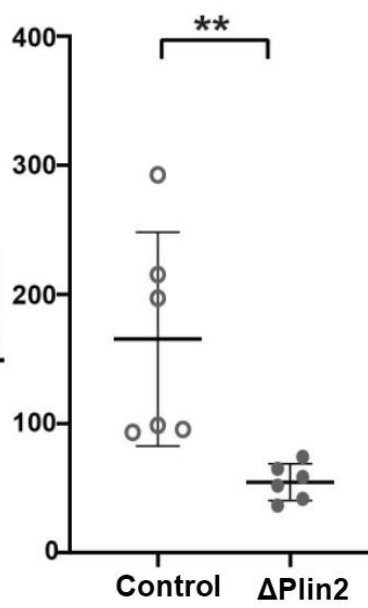

Supplement: Supp_Fig_8_zjae195 [file supp_fig_8_zjae195.pdf]
