# Supplementary material for: Enhanced fatty acid oxidation in osteoprogenitor cells provides protection from high-fat diet induced bone dysfunction
Source: J Bone Miner Res. 2024 Dec 8;40(2):283–98. doi: 10.1093/jbmr/zjae195 (PMC11789392; doi:10.1093/jbmr/zjae195)
Supplement: Supp_Table_1_zjae195 [file supp_table_1_zjae195.pdf]

Supplemental Table 1. qRT-PCR Primer Sequences.

Gene	Primer Sequence (5'-3')	
Pnpla2	FWD:	GAGTGCAGTGTCCTTCACCA
	REV:	ATCAGGCAGCCACTCCAAC
Lipe	FWD:	GCTGGACTGTCAAGCACTGT
	REV:	GTAACTGGGTAGGCTGCCAT
Mgll	FWD:	GACACCATCCAGAAGGACTACC
	REV:	GATTGGCAAGGACCAGAGGTGA
Lipa	FWD:	GAGTTCTGGGCCTTCAGTT
	REV:	GCCTTGAGAATGACCCACATA
Cpt1a	FWD:	TCCAGTTGGCTTATCGTGGTG
	REV:	CTAACGAGGGGTCGATCTTGG
Cpt2	FWD:	CCTCGCTCAGGATAAACA
	REV:	GTGTCTTCAGAAACCGCACTG
Mfn2	FWD:	CTGCTAGGAGTTGCTGCATATAA
	REV:	TCAGCCATGTGTCGCTTATC
Plin5	FWD:	GCAGCTTCTCTTCCAATTTGTC
	REV:	GTGTGTAGTGTGACTACCTGTG
Plin2	FWD:	TTGAGATCCTGTGTGAGATG
	REV:	CCCTTGCAGGCATAGGTATT
Hprt1	FWD:	GCCTAAGATGAGCGCAAGTTG
	REV:	TACTAGGCAGATGGCCACAGG
