# Supplementary material for: Enhanced fatty acid oxidation in osteoprogenitor cells provides protection from high-fat diet induced bone dysfunction
Source: J Bone Miner Res. 2024 Dec 8;40(2):283–98. doi: 10.1093/jbmr/zjae195 (PMC11789392; doi:10.1093/jbmr/zjae195)
Supplement: Supp_Table_2_zjae195 [file supp_table_2_zjae195.pdf]

Supplemental Table 2. Free long-chain fatty acid tandem mass spectrometric detection.

	Parent	Center	Width	Time	CE	Q1 PW	Q3 PW	Tube Lens
C6:0	230.1	171.0	1.5	0.015	15	0.7	0.7	112
C6:0	230.1	100.1	1.5	0.015	25	0.7	0.7	112
C8:0	258.1	100.1	1.5	0.015	20	0.7	0.7	112
C8:0	258.2	199.2	1.5	0.015	15	0.7	0.7	112
C10:0	286.1	227.2	1.5	0.015	17	0.7	0.7	121
C10:0	286.1	100.1	1.5	0.015	22	0.7	0.7	121
C12:0	314.1	255.0	1.5	0.015	17	0.7	0.7	122
C12:0	314.1	100.1	1.5	0.015	23	0.7	0.7	122
C14:0	342.1	283.4	1.5	0.015	17	0.7	0.7	130
C14:0	342.1	100.1	1.5	0.015	23	0.7	0.7	130
C16:2	366.1	307.1	1.5	0.015	21	0.7	0.7	123
C16:2	366.1	100.1	1.5	0.015	25	0.7	0.7	123
C16:1	368.1	309.1	1.5	0.015	21	0.7	0.7	123
C16:1	368.1	100.1	1.5	0.015	25	0.7	0.7	123
C16:0	370.1	311.0	1.5	0.015	21	0.7	0.7	123
C16:0	370.1	352.0	1.5	0.015	19	0.7	0.7	123
C16:0	370.1	100.1	1.5	0.015	25	0.7	0.7	123
C16:0-d4	374.1	315.0	1.5	0.015	20	0.7	0.7	149
C16:0-d4	374.1	257.0	1.5	0.015	25	0.7	0.7	149
C16:0-d4	374.1	100.1	1.5	0.015	25	0.7	0.7	149
C18:2	394.0	335.0	1.5	0.015	19	0.7	0.7	151
C18:2	394.1	376.0	1.5	0.015	22	0.7	0.7	151
C18:2	394.1	100.1	1.5	0.015	26	0.7	0.7	151
C18:1	396.1	337.1	1.5	0.015	20	0.7	0.7	145
C18:1	396.1	100.1	1.5	0.015	25	0.7	0.7	145
C18:0	398.1	339.0	1.5	0.015	23	0.7	0.7	145
C18:0	398.1	281.0	1.5	0.015	18	0.7	0.7	145
C18:0	398.1	100.1	1.5	0.015	27	0.7	0.7	145
C20:2	422.1	363.1	1.5	0.015	23	0.7	0.7	126
C20:2	422.1	100.1	1.5	0.015	25	0.7	0.7	126
C20:1	424.1	365.1	1.5	0.015	23	0.7	0.7	126
C20:1	424.1	100.1	1.5	0.015	25	0.7	0.7	126
C20:0	426.1	310.0	1.5	0.015	25	0.7	0.7	126
C20:0	426.1	367.0	1.5	0.015	23	0.7	0.7	126
C20:0	426.1	100.1	1.5	0.015	28	0.7	0.7	126
C22:0	454.1	100.1	1.5	0.015	25	0.7	0.7	150
C22:0	454.1	395.0	1.5	0.015	20	0.7	0.7	150

C22:0-d4	458.1	399.0	1.5	0.015	20	0.7	0.7	150
C22:0-d4	458.1	100.1	1.5	0.015	25	0.7	0.7	150
C24:0	482.1	423.0	1.5	0.015	20	0.7	0.7	150
C24:0	482.1	100.1	1.5	0.015	25	0.7	0.7	150
C24:0-d4	486.1	427.0	1.5	0.015	20	0.7	0.7	150
C24:0-d4	486.1	100.1	1.5	0.015	25	0.7	0.7	150
