# Supplementary material for: Enhanced fatty acid oxidation in osteoprogenitor cells provides protection from high-fat diet induced bone dysfunction
Source: J Bone Miner Res. 2024 Dec 8;40(2):283–98. doi: 10.1093/jbmr/zjae195 (PMC11789392; doi:10.1093/jbmr/zjae195)
Supplement: Supp_Table_3_zjae195 [file supp_table_3_zjae195.pdf]

Supplemental Table 3. Acylcarnitine Tandem Mass Spectrometric Detection.

Acylcarnitine	Accurate m/z	mass error, ppm	Linear range, ng/mL
C16:0	400.3421	2.34	0.25-100
C16:0 (1,2,3,4- 13C4) ISTD	404.3556	1.58	NA
C18:0	428.3734	2.04	1-100
C18:1	426.3578	2.22	0.25-100
C18:2	424.3421	2.46	0.25-100
