# Supplementary material for: Enhanced fatty acid oxidation in osteoprogenitor cells provides protection from high-fat diet induced bone dysfunction
Source: J Bone Miner Res. 2024 Dec 8;40(2):283–98. doi: 10.1093/jbmr/zjae195 (PMC11789392; doi:10.1093/jbmr/zjae195)
Supplement: Supp_Figure_Legends_Clean_zjae195 [file supp_figure_legends_clean_zjae195.docx]

**Supplementary Figure Legends**

**Figure S1.** Additional µCT parameters from male mice fed a control (Con) or high fat diet (HFD**)** to include (A) connectivity density (Conn.D) and (B) structural model index (SMI) of the distal femur metaphysis. Cortical bone properties from the femur mid-diaphysis include (C) medullary volume (Ma.V) and (D) minimum polar moment of inertia (MMOI). Each dot represents data from individual animals (*n*=6-10). Statistical comparisons are between the two groups using unpaired t-tests. All results are expressed as mean ± standard deviation. **p* < 0.05, ***p* < 0.01, ****p* < 0.001, *****p* < 0.0001.

**Figure S2.** Raw image of thin-layer chromatogram (TLC) of lipids harvested from flushed tibias following 8 weeks on a control (Con) and high fat diet (HFD) along with standards.

**Figure S3.** Quantification of Plin2 expression in (A) flushed femur cortex from Cre-negative Plin2^fl/fl^ littermate controls (Control) and Prrx1-Cre+. Plin2^fl/fl^ (ΔPlin2) mice normalized to total protein. (B) Quantitative real time PCR of *Plin2* in *ex vivo* differentiated osteoblasts from BMSCs from control and ΔPlin2 mice normalized to the internal housekeeping gene, *Hprt1*. Statistical comparisons are between the two groups using unpaired t-tests. Data are means ± standard deviation where *, p <0.05, **, p <0.01, ***, p <0.001, ****, p <0.0001.

**Figure S4.** Additional **s**erum lipid profile of (A) high-density lipoprotein (HDL) (*p-value: genotype = 0.5502; diet = <0.0001*), (B) triglyceride (*p-value: genotype = 0.3935; diet = 0.0108*), and (C) non-esterified fatty acids (NEFA) (*p-value: genotype = 0.8919; diet = 0.8344*) from male control or ΔPlin2 mice fed a control (ConDiet) or high fat diet (HFD) for 12 weeks. Each dot represents data from an individual animal, (*n*=9-11). All results are expressed as mean ± standard deviation.  Significant differences were established using 2-way analyses of variance (2-way ANOVA) with genotype and diet as independent variables, with post-hoc uncorrected Fisher’s LSD tests. Values of p < 0.05 were considered significant, with p-values depicted as **p* < 0.05, ***p* < 0.01, ****p* < 0.001, *****p* < 0.0001.

**Figure S5.** Characterization from female control or ΔPlin2 mice fed a control (ConDiet) or high fat diet (HFD) for 12 weeks to include (A) final bodyweight (*p-value: genotype = 0.3690; diet = <0.0001*); (B) fat weight (*p-value: genotype = 0.3192; diet = <0.0001*); (C) lean weight (*p-value: genotype = 0.0902; diet = <0.7111*); (D) glucose tolerance test (GTT) area under the curve (*p-value: genotype = 0.0111; diet = <0.0001*); and (E) serum cholesterol (*p-value: genotype = 0.7937; diet = 0.0023*). Each dot represents data from an individual animal, (*n*=6-10). Significant differences were established using 2-way analyses of variance (2-way ANOVA) with genotype and diet as independent variables, with post-hoc uncorrected Fisher’s LSD tests. Values of p < 0.05 were considered significant, with p-values depicted as **p* < 0.05, ***p* < 0.01, ****p* < 0.001, *****p* < 0.0001.

**Figure 6S.** Additional µCT parameters of the femur from male control or ΔPlin2 mice fed a control (ConDiet) or high fat diet (HFD) to include cortical (A) minimum polar moment of inertia (MMOI) (*p-value: genotype = 0.6882; diet = 0.0095*) and (B) thickness (Ct.Th) (*p-value: genotype = 0.5389; diet = 0.9160*). (C) Trabecular bone volume per total volume (BV/TV) of the L6 vertebra (*p-value: genotype = 0.3440; diet = 0.0090*). Each dot represents data from an individual animal, (*n*=8-11). Significant differences were established using 2-way analyses of variance (2-way ANOVA) with genotype and diet as independent variables, with post-hoc uncorrected Fisher’s LSD tests. Values of p < 0.05 were considered significant, with p-values depicted as **p* < 0.05, ***p* < 0.01, ****p* < 0.001, *****p* < 0.0001.

**Figure S7.** Trabecular µCT parameters from female control or ΔPlin2 mice fed a control (ConDiet) or high fat diet (HFD). Parameters include distal femur metaphysis (A) bone volume per total volume (BV/TV) (*p-value: genotype = 0.8543; diet = 8507*); (B) trabecular number (Tb.N) (*p-value: genotype = 0.8826; diet = 2768*); (C) trabecular thickness (Tb.Th) (*p-value: genotype = 5767; diet = 1788*); and (D) trabecular separation (Tb.Sp) (*p-value: genotype = 0.9774; diet = 0.0743*). (E) Trabecular bone was also analyzed in the L6 vertebra and BV/TV is reported (*p-value: genotype = 0.4280; diet = 0.2531*). Each dot represents data from an individual animal, (*n*=7-10). Significant differences were established using 2-way analyses of variance (2-way ANOVA) with genotype and diet as independent variables, with post-hoc uncorrected Fisher’s LSD tests. Values of p < 0.05 were considered significant, with p-values depicted as **p* < 0.05, ***p* < 0.01, ****p* < 0.001, *****p* < 0.0001.

**Figure S8.** (A) Representative confocal images from bone marrow stromal cells (BMSCs) following 8 days in osteogenic differentiation, isolated from control or ΔPlin2 mice. Cellular lipid droplets were stained with BODIPY 493/503 (green in merged panel). Quantification of (B) intensity and the of BODIPY 493/503-stained lipid droplets in differentiated osteoblasts from control or ΔPlin2 mice where each dot represents the size or intensity of one lipid droplet. (C) Quantification of the number of lipid droplets per cell in differentiated osteoblasts from control and ΔPlin2 mice. Data are means ± standard deviation from *n*=3 mice from each group. Statistical comparisons are between the two groups using unpaired t-tests. Values of p < 0.05 were considered significant, with p-values depicted as **p* < 0.05, ***p* < 0.01, ****p* < 0.001, *****p* < 0.0001.
